# Next-generation approach to skin disorder prediction employing hybrid deep transfer learning

**DOI:** 10.3389/fdata.2025.1503883

**Published:** 2025-02-19

**Authors:** Yonis Gulzar, Shivani Agarwal, Saira Soomro, Meenakshi Kandpal, Sherzod Turaev, Choo W. Onn, Shilpa Saini, Abdenour Bounsiar

**Affiliations:** ^1^Department of Management Information Systems, College of Business Administration, King Faisal University, Al-Ahsa, Saudi Arabia; ^2^Department of Information Technology, Ajay Kumar Garg Engineering College, Ghaziabad, India; ^3^Department of Distance Continuing and Computer Education, Faculty of Education, University of Sindh, Jamshoro, Pakistan; ^4^Computer Science and Engineering, Odisha University of Technology and Research, Bhubaneswar, India; ^5^Department of Computer Science and Software Engineering, College of Information Technology, United Arab Emirates University, Al Ain, United Arab Emirates; ^6^Faculty of Data Science and Information Technology, INTI International University, Persiaran Perdana BBN, Putra Nilai, Nilai Negri Sembilan, Malaysia; ^7^Department of Computer Science and Engineering, Chandigarh University, Mohali, India; ^8^Department of Computer Science, College of Computer Sciences and Information Technology, King Faisal University, Al Hofuf, Saudi Arabia

**Keywords:** skin disorder prediction, deep learning, transfer learning, DenseNet121, EfficientNetB0, computer vision, image classification

## Abstract

**Introduction:**

Skin diseases significantly impact individuals' health and mental wellbeing. However, their classification remains challenging due to complex lesion characteristics, overlapping symptoms, and limited annotated datasets. Traditional convolutional neural networks (CNNs) often struggle with generalization, leading to suboptimal classification performance. To address these challenges, this study proposes a Hybrid Deep Transfer Learning Method (HDTLM) that integrates DenseNet121 and EfficientNetB0 for improved skin disease prediction.

**Methods:**

The proposed hybrid model leverages DenseNet121's dense connectivity for capturing intricate patterns and EfficientNetB0's computational efficiency and scalability. A dataset comprising 19 skin conditions with 19,171 images was used for training and validation. The model was evaluated using multiple performance metrics, including accuracy, precision, recall, and F1-score. Additionally, a comparative analysis was conducted against state-of-the-art models such as DenseNet121, EfficientNetB0, VGG19, MobileNetV2, and AlexNet.

**Results:**

The proposed HDTLM achieved a training accuracy of 98.18% and a validation accuracy of 97.57%. It consistently outperformed baseline models, achieving a precision of 0.95, recall of 0.96, F1-score of 0.95, and an overall accuracy of 98.18%. The results demonstrate the hybrid model's superior ability to generalize across diverse skin disease categories.

**Discussion:**

The findings underscore the effectiveness of the HDTLM in enhancing skin disease classification, particularly in scenarios with significant domain shifts and limited labeled data. By integrating complementary strengths of DenseNet121 and EfficientNetB0, the proposed model provides a robust and scalable solution for automated dermatological diagnostics.

## 1 Introduction

Skin diseases affect millions of people globally, affecting both physical and mental health. These conditions range from minor infections to severe chronic illnesses and encompass infections, inflammatory responses, chronic conditions, and hereditary disorders. The symptoms—such as rashes, itching, discoloration, dryness, and texture changes—can vary widely in severity and are often influenced by genetics, immune responses, environmental factors, and lifestyle choices (Li et al., 2021; Meedeniya et al., 2024). This diversity not only makes conditions hard to distinguish visually but also means that early symptoms are often similar across different disorders, leading to frequent misdiagnosis. As a result, the accurate identification of skin diseases presents significant challenges, especially in clinical settings where visual assessments are the primary diagnostic method. Effective management generally involves medical intervention, lifestyle modification, and preventive care (Alshahrani et al., 2024). Given the large global population affected, skin diseases are a significant public health concern (Behara et al., 2024).

Early diagnosis is essential for both prevention and effective treatment, but the visual complexity of many skin conditions often makes accurate diagnosis challenging, which can delay treatment and increase risks. In some cases, late diagnosis may lead to severe outcomes, including skin cancer (Gulzar and Khan, 2022; Mehmood et al., 2023). The importance of precise and early identification for reducing morbidity and mortality rates underscores the need for ongoing dermatological research (Chan et al., 2020).

Skin disorders are caused by a complex interplay of genetic, immunological, and environmental factors. As the body's largest organ, the skin acts as a protective barrier; however, it is highly susceptible to environmental stressors such as ultraviolet (UV) radiation, chemicals, and pathogens (Inthiyaz et al., 2023). Chronic skin diseases such as psoriasis and vitiligo are frequently associated with genetic predispositions (Abdallah et al., 2023), whereas immune responses play a critical role, as autoimmune reactions can cause inflammation and weakened immunity may heighten infection risks (Bucsek et al., 2018). Recent research on the skin microbiome has revealed its essential role in barrier function and infection prevention, adding another dimension to the understanding of skin health (Harris-Tryon and Grice, 2022).

Traditional diagnostic methods often rely on subjective visual assessments, which can lead to inaccuracies, especially with the diverse presentations and overlapping symptoms of skin diseases. Artificial intelligence (AI) has been used in diverse domains, including agriculture (Gulzar, 2024; Amri et al., 2024; Gulzar et al., 2023b), finance (Gulzar et al., 2023a), healthcare (Gulzar and Khan, 2022; Mehmood et al., 2023), and environmental monitoring (Malik et al., 2023), to address complex challenges and improve accuracy in decision-making processes. In dermatology, AI has emerged as a powerful tool for enhancing diagnostic precision. AI-powered image recognition algorithms can detect skin abnormalities such as eczema, psoriasis, and melanoma, offering early detection that improves patient prognosis and accessibility, particularly through tools like digital dermatoscopes and smartphone apps (Sengupta, 2023; Ye and Chen, 2023). AI also enables personalized treatment plans by analyzing patient-specific factors like medical history, genetics, and lifestyle to predict treatment efficacy and minimize adverse effects, especially for chronic conditions (Khan et al., 2023a; Anand et al., 2023; Khan et al., 2023b). In telemedicine, AI-based applications facilitate remote consultations and triage, reducing wait times and prioritizing urgent cases (Majid et al., 2023a,b). Additionally, AI contributes to drug discovery by identifying potential treatments and optimizing regimens for conditions like dermatitis, psoriasis, and acne (Rokni et al., 2024), ultimately advancing evidence-based, individualized dermatological care (Meedeniya et al., 2024; Jain et al., 2024).

Recent advancements have shown that AI can further elevate the precision and effectiveness of skin disease management. By leveraging cutting-edge architectures, AI-based models can address the inherent challenges in classifying skin diseases, such as their visual complexity, overlapping symptoms, and variations in lesion patterns. Traditional Convolutional Neural Network (CNN) models often fall short in generalizing to new or diverse datasets, particularly when labeled data are limited. This study proposes a Hybrid Deep Transfer Learning Model (HDTLM) that combines DenseNet and EfficientNet architectures. DenseNet, known for its dense connections, captures fine-grained features, whereas EfficientNet fine-tunes these features, thereby improving computational efficiency without compromising performance. Furthermore, domain adversarial training was employed to ensure that the learned features remain relevant across different datasets, which improves the model's generalizability and robustness in real-world applications.

The proposed model was rigorously tested on various benchmark datasets, achieving a training accuracy of 98.18% and a validation accuracy of 86.68%, outperforming traditional transfer learning methods. This approach not only demonstrates the potential of AI to enhance diagnostic accuracy and reduce overfitting but also provides a promising solution for domains with limited labeled data and significant variability. By integrating AI into dermatological research, this study seeks to address key issues in skin disease classification and prediction, aiming for a scalable model that adapts to diverse clinical settings.

The contributions of this study include:

- Introducing a Hybrid Deep Transfer Learning Model (HDTLM) combining DenseNet and EfficientNet, optimized for skin disease classification.
- High training and validation accuracy surpassing traditional transfer learning methods in performance and robustness.
- Comprehensive data preprocessing techniques, including augmentation and normalization, to improve model generalizability with limited labeled data.
- Thorough benchmarking on multiple skin disease datasets to validate improved accuracy, precision, and reduced overfitting.
- Insights into overcoming challenges such as domain shifts, data imbalance, and hyperparameter tuning specific to skin disease classification tasks.

The remainder of this study is organized as follows. In Section Related work, the related works relevant to this study are reviewed and discussed. Materials and methods are detailed in Section Material and methods. Section Results and discussion describes the results and discusses the findings. This study has limitations, which are discussed in section Limitations and future work along with possible directions for future work. Section Conclusion presents the conclusion.

## 2 Related work

### 2.1 Challenges in skin disease diagnosis

Skin diseases pose significant challenges in healthcare because of their diverse appearances and the need for accurate, specific diagnoses. Traditional diagnostic methods rely heavily on dermatologists' expertise, which can be subjective, time-consuming, and resource-intensive. These challenges have driven the increasing adoption of deep learning techniques to automate skin disease classification, aiming to enhance diagnostic accuracy and efficiency. To address these challenges, numerous studies have explored the use of deep learning techniques for automating skin disease classification.

### 2.2 Application of deep learning in skin disease classification

Several studies have explored the application of deep learning in skin disease classification. For instance, De et al. (2024) automatically identified skin diseases using dermatoscopic imaging. The authors also mentioned that, traditionally, dermatologists manually examined pigmented skin lesions, which can be subjective and time-consuming. Agarwal and Godavarthi (2023) evaluated and compared various skin diseases in terms of cosmetics and common skin issues. The author's dataset included ~25,000 records for the eight most common skin conditions, and a convolutional neural network was used to achieve imaging performance comparable to or superior to that of humans. Naeem et al. (2024) proposed SNC_Net, which applies deep learning models that include features from dermoscopic images and handmade (HC) feature extraction approaches to improve classifier performance. Alshahrani et al. (2024) combined CNN models (DenseNet121, MobileNet, and VGG19) with Random Forest (Rf) and Feed Forward Neural Networks (FFNN) networks to obtain complex features from dermoscopy images, and the result was a hybrid system capable of early detection of various skin lesions. Behara et al. (2024) employed adaptive thresholding to extract regions of interest (ROI) and improved cancer detection accuracy through dynamic capabilities. Khan et al. (2021) employed the PAD-UFES-20 dataset, which includes six different types of imbalanced skin cancer types, to address the data imbalance using data augmentation. Wei et al. (2023) presented a convolutional neural network model for skin disease categorization using model fusion. Sharma et al. (2023) analyzed the HAM10000 dataset to assess the profitability of several Convolutional Neural Network (CNN) designs in concealing seven different types of skin lesions. In this study, we employed EfficientNets, which outperforms standard designs because of its lightweight design. Venugopal et al. (2023) implemented improved EfficientNet B4 and EfficientNet V2-M models to categorize malignant and benign skin lesions in dermoscopic images. Adegun and Viriri (2020) proposed a framework to segment and classify skin lesions to automatically detect skin cancer. The suggested structure of this problem is divided into two parts. The basic part of this network is used to analyze mixed and complex data problems. In this step, the algorithm acquires the surrounding details using decryption. Ravi (2022) proposed classifying and detecting learning-based mixed features grouped for skin cancer that consider attention costs. Shimu et al. (2022) proposed the implementation of transfer learning to six forms of skin diseases: peeling, acne, eczema, heat rash, melanoma, and cold sores. The skin conditions were classified using a Convolutional Neural Network. Sadik et al. (2023) focused on employing CNNs to become adept at skin disease recognition, similar to the renowned detective Sherlock Holmes. They wanted to know how well these architectures could perform the job. AlSuwaidan (2023) compared six popular CNNs (VGG16, EfficientNet, InceptionV3, MobileNet, NasNet, and ResNet50) to determine which one was the best at guessing the top three skin issues in the Middle East. They cleaned up the images a bit with some filtering and denoising to ensure the models had the best view. Jaisakthi et al. (2023) developed a Deep Convolutional Neural Network (DCNN)-based model to cartegorize skin cancer into melanoma and non-melanoma. Milantev et al. (2020) conducted the ISIC Skin Lesion Classification Challenge using dermoscopic images and patient information to study skin lesions. Tahir et al. (2023) developed DSCC_Net, which uses a CNN to classify skin cancer and evaluates it on three big datasets (ISIC 2020, HAM10000, and DermIS). Gupta et al. (2020) presented a system that employs transfer learning with pretrained models to improve the results. Furthermore, this study used a CNN to precisely detect and categorize skin cancer. Pham et al. (2020) suggested a mixed strategy to address class disparities in skin disease categorization. This process combined data-level balanced mini-batch logic with real-time image augmentation and algorithm-level generation of new loss functions. Abbasi et al. (2024) proposed a modified VGG16-based algorithm to distinguish real and AI-generated medical images. The model was trained and fine-tuned using hyperparameter tuning on a dataset of 10,000 synthetic skin lesion images produced by a GAN. It distinguished real images from AI-generated images with 99.82% accuracy. Gamage et al. (2023) trained an Xception-based model for melanoma classification using the HAM10000 dataset. Bayesian hyperparameter optimization was employed alongside Grad-CAM/Grad-CAM++ heatmaps for improved explainability, where the model reached an accuracy of 90.24% while providing critical input regions that influence model predictions.

### 2.3 Summary of related work

Table 1 summarizes the key contributions, findings, and limitations of recent studies. These studies highlight the significant progress made in applying deep learning to skin disease classification while also identifying areas for improvement, such as addressing overfitting, improving data diversity, and ensuring computational efficiency.

**Table 1:** Contributions and limitations of recent skin disease classification studies.

**References**	**Main contribution**	**Key findings**	**Limitations**
De et al. (2024)	Apply deep CNN techniques to determine and classify skin diseases.	Using deep learning models, including CNNs, there was successful recognition and classification of various skin diseases.	Validating the system's efficacy in clinical trials and comparing it to dermatologist expertise are crucial for ensuring dependability and safety.
Agarwal and Godavarthi (2023)	Comparing and contrasting different skin diseases from the viewpoint of cosmetics and common skin issues.	ResNet152 outperformed other deep learning algorithms on a 1,930 picture dataset, achieving higher recall, accuracy, and precision.	The dataset has an unequal distribution of images for each type of skin disorder, as well as variances in image illumination and commonalities among skin ailments.
Naeem et al. (2024)	CNN was applied for sorting.	CNN was employed to categorize the illnesses, while HC and the Inception v3 (DL method) extracted key features from the dermoscopy images.	A larger dataset is required to evaluate the proposed model accurately.
Alshahrani et al. (2024)	Enhance the AI system's potential to achieve selectivity and resistance.	For detecting early skin cancer, this model helps generate mixed features and collect information from different sources.	Lower accuracy in offline data prediction.
Behara et al. (2024)	Grid-based, understandable design and size of the CNN were designed to accurately and interpretably classify skin cancer.	This research offers automated technology for dermatological diagnostics that can be used to detect skin cancer using a tool.	The model achieved a computing time of 0.55 s, which is far faster than that of previous methods.
Khan et al. (2021)	To develop a machine-based diagnostic system for skin cancer and evaluate the model to investigate clinical factors in the diagnosis that improve outcomes from previous studies.	The results of the proposed investigation verified Pacheco's findings that integrating clinical data improves diagnosis and triage effectiveness.	The suggested model must be validated on numerous datasets.
Wei et al. (2023)	The focus element for extracting features was improved by incorporating model-wide and broad feature fusion and an attention module.	To collect the features of wide and broad layers by applying the parallel technique.	The suggested approach consumed an extensive amount of CPU resources during training.
Sharma et al. (2023)	Showing outperformance of models, ResNets and VGG16, in terms of precision and recall despite their lightweight design.	The EfficientNet model improved accuracy without requiring extensive preprocessing or data augmentation procedures.	The dataset was heavily unbalanced.
Venugopal et al. (2023)	For multi-class and binary classification judged by training models.	To accelerate performance and overcome overfitting using transfer learning and data augmentation.	The rate of training is slow.
Adegun and Viriri (2020)	FCN used skip routes with extended and short-cut connections and did not prefer typical FCNs that solely employ long skip connections.	By showing how the segmentation network affects the classification of unsegmented images.	Low performance with limited data.
Ravi (2022)	By introducing the learning model for detecting the accuracy and classifying the skin disorders based on images.	The results of this study gave greater weight to classes with some skin disease datasets and classes with a high number of samples.	It is possible that certain functionalities will be lost during this stage.
Shimu et al. (2022)	Four advanced transfer learning models, including NASNetLarge, InceptResNetV2, EfficientNetB1, and DenseNet169, were compared to CNN.	The suggested approach relied heavily on the CNNs and transfer learning models for illness classification.	The research's key weakness is excessive variability caused by overfitting.
Sadik et al. (2023)	Using CNN architectures, MobileNet, and Xception, an expert system can recognize several skin conditions with high accuracy and efficiency.	These approaches could improve dermatological disease recognition and diagnosis and assist healthcare professionals in providing enhanced treatment.	Not applicable to diverse datasets.
AlSuwaidan (2023)	DL architectures rely heavily on data, and some studies have used picture augmentation to enhance the number of images.	This study used image filtering and denoising for BM3D noise reduction and edge improvement.	Limited dermatological disorders.
Jaisakthi et al. (2023)	The study employed datasets with known challenges, which have varying image resolutions and class imbalance difficulties.	Applied background data and trained data by using a classifier to extract features from the layers.	Lower accuracy in offline data prediction.
Milantev et al. (2020)	For training datasets of 25,331 images from 8 distinguished classes and undefined classes.	Apply preprocessed metadata to create thick layers in the ensemble.	The unnecessary parts of the image were not removed.
Tahir et al. (2023)	There are four different types of skin cancer and blocks of conventional layers in DSCC_Net to identify pre-stage cancers.	The updated structure's convolutional blocks used numerous layers to identify the pre-staged skin tumors.	The proposed DSSC_Net model can be used to identify the disadvantages of fair skin.
Gupta et al. (2020)	This paper proposed using the HAM10000 dataset to classify skin cancer into seven groups and identify a methodology that includes image processing and deep learning models.	The DenseNet, ResNet50, and Inception_V3 learning provide the outputs of efficient Net B1 classification.	The model was disadvantaged by DenseNet and EfficientNet B1 due to the imbalanced dataset.
Pham et al. (2020)	Six proposed approaches' performance indicators were examined on a test dataset of 2,453 images.	When implemented in both CNN architectures, CLF outperformed both ORI and BON in terms of mRecall.	Not applicable to multiple skin disease classifications, medical image analysis, and common imbalanced datasets.

## 3 Material and methods

**Figure 1:**
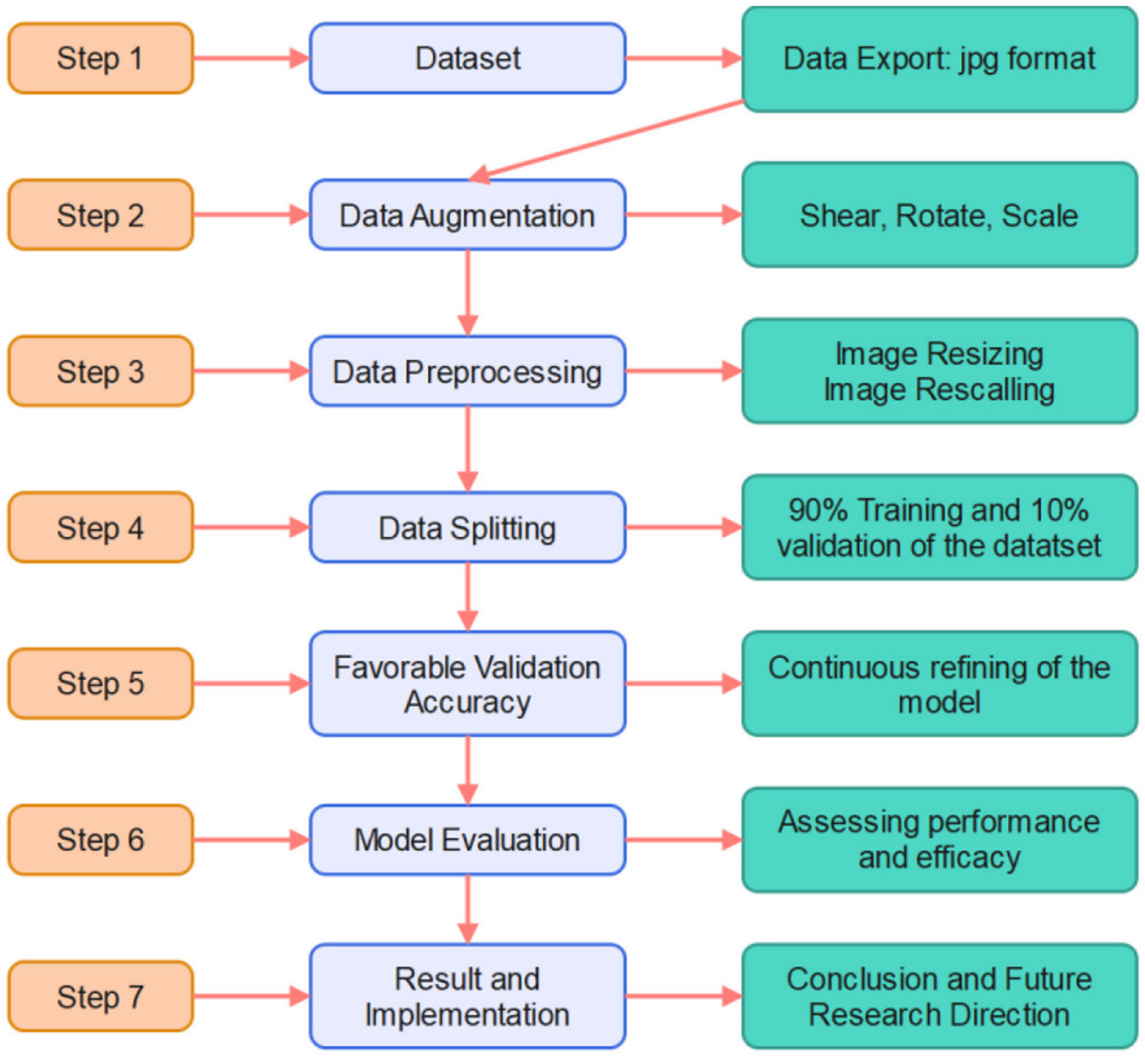
Research framework.

As shown in Figure 1, the development of a skin disease prediction model using transfer learning with DenseNet121 and EfficientNetB0 required a structured, methodical approach. This approach encompassed multiple stages, including data collection, data preprocessing, data augmentation, model selection, model training, evaluation, and comprehensive analysis. Each step was meticulously executed to ensure robustness and reliability in predicting skin diseases across various categories. This section provides a detailed breakdown of the research methodology employed in constructing the model, emphasizing the integration of transfer learning to enhance model generalization and performance on limited domain-specific data.

### 3.1 Dataset and data augmentation

**Figure 2:**
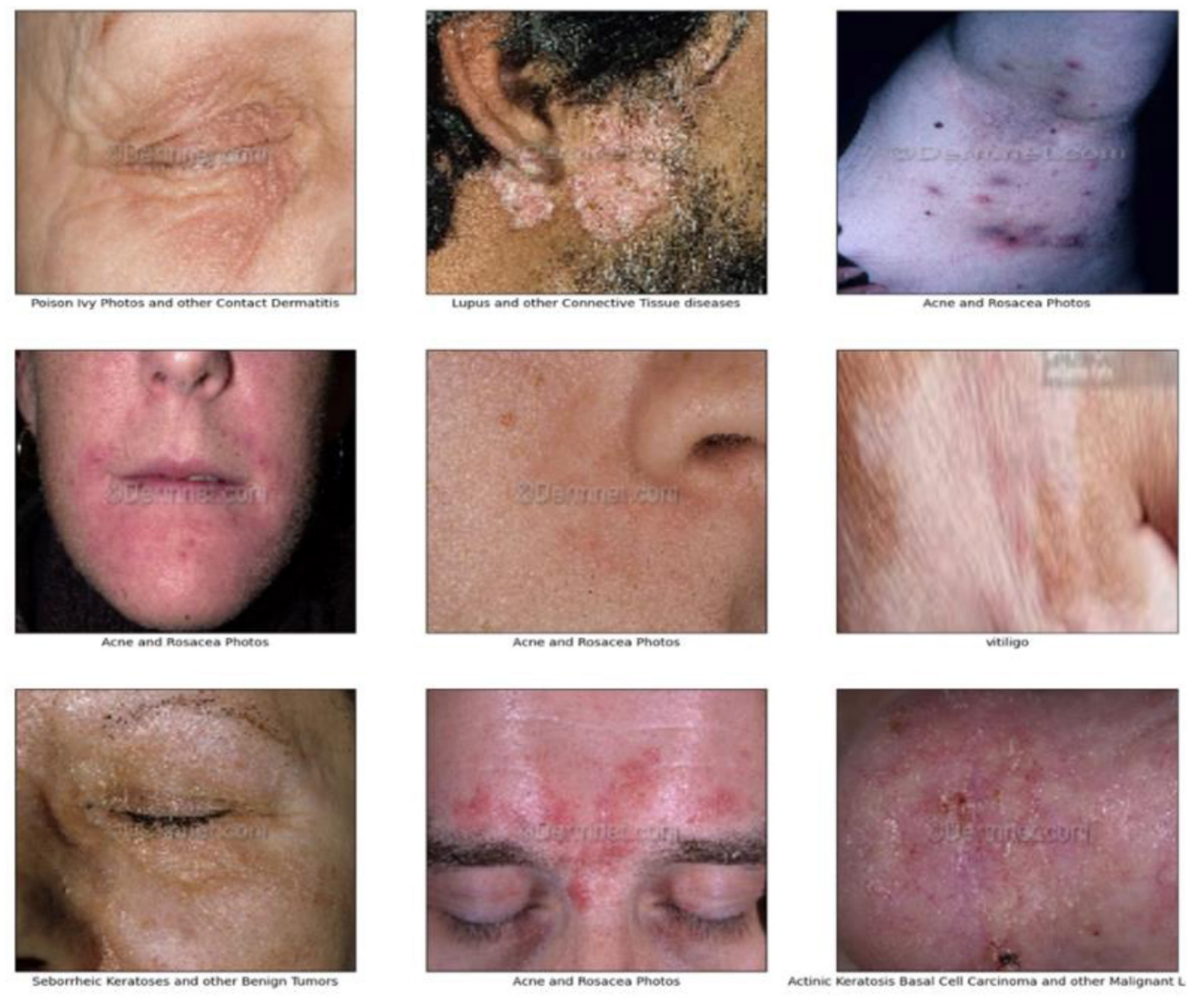
Sample images of the dataset.

Following a thorough review of existing skin disease datasets, we selected a public resource that provides extensive coverage of 19 skin conditions (Skin Diseases Dataset, 2024),. including Vitiligo, Psoriasis, Acne, Actinic Carcinoma, Atopic Dermatitis, Cellulitis, Eczema, Drug Eruptions, Herpes HPV, Light Diseases, Lupus, Melanoma, Poison Ivy, Benign Tumors, Systemic Disease, Ringworm, Urticarial Hives, Vascular Tumors, Vasculitis, and Viral Infections. Each condition was represented by sufficient images for both training and testing, thereby enhancing the dataset's applicability to dermatological research. Figure 2 shows sample images from the dataset, demonstrating its diversity.

We employed a systematic and targeted data augmentation strategy to enhance the robustness and generalizability of the dataset. This approach improved the diversity and representativeness of the dataset while addressing class imbalances in underrepresented categories.

Image augmentations included random 30-degree rotations, zooms in or out by 20%, and 10%-20% horizontal or vertical shifts. These augmentations represented the common real-world variation in image posture, scale, and location, facilitating a broadly varied dataset that accurately depicts realistic conditions (Ayoub et al., 2023). We also applied horizontal flipping to add more variance. These augmentation techniques were implemented by utilizing TensorFlow's ImageDataGenerator to implement real-time augmentation during the preparation of the dataset. This not only maintained the original image quality but also allowed for uniform expansion of the dataset.

Particular focus was given to classes where the number of original images was limited, specifically classes with 5, 16, 37, 26, 29, and 46 samples. For these classes, the augmentation process was repeated numerous times by using different combinations of transformations to accrete a much larger dataset. This allowed a more proportionate representation of each class, reducing the bias and creating a more robust diversity in the overall dataset. This ensured that the dataset was broadening its representativeness of clinical variability found in the real world by generating more samples for rare conditions, which made analyses performed later much more robust. Table 2 presents the number of images per class before and after augmentation and divides the augmented dataset into training, validation, and testing subsets based on a 70:15:15 ratio.

**Table 2:** Number of images per class before and after data augmentation.

**Classes**	**#Images before augmentation**	**#Images after augmentation**	**Train (70%)**	**Validation (15%)**	**Test (15%)**
Acne	1,071	1,071	750	161	160
Actinic carcinoma	442	1,020	714	153	153
Atopic dermatitis	124	1,018	713	153	152
Cellulitis	79	1,010	707	152	151
Eczema	37	1,000	700	150	150
Drug eruptions	26	1,000	700	150	150
Herpes HPV	46	1,010	707	152	151
Light diseases	329	1,050	735	158	157
Lupus	115	1,010	707	152	151
Melanoma	29	1,000	700	150	150
Poison IVY	75	1,020	714	153	153
Psoriasis	86	1,020	714	153	153
Benign tumors	111	1,010	707	152	151
Systemic disease	108	1,020	714	153	153
Ringworm	164	1,000	700	150	150
Urticarial hives	5	1,000	700	150	150
Vascular tumors	138	1,000	700	150	150
Vasculitis	16	1,010	707	152	151
Viral infections	299	1,020	714	153	153

### 3.2 Data preprocessing

To standardize the dataset for consistent model input, several preprocessing steps were applied. Images were resized to a uniform 128 × 128 pixels, and color values were adjusted to ensure consistency across samples. Pixel values were normalized to a 0–1 scale, improving training stability and convergence speed. The labels were one-hot encoded to format them appropriately for classification tasks, thereby enabling the model to distinguish between different classes effectively (Ayoub et al., 2022).

For model evaluation, the dataset was divided into training, validation, and testing sets, with 70% of the data allocated to training and 15% each for validation and testing. This balanced split supports robust model training, validation, and testing, ensuring generalizability across unseen data.

### 3.3 Proposed model

The proposed model for skin disease prediction combines the strengths of DenseNet121 and EfficientNetB0 through a hybrid transfer learning approach. Each architecture was selected for its unique capabilities, resulting in a robust model that could handle the complexity and variability of skin disease images.

- **DenseNet121:** Known for its dense connections, this architecture reuses features by directly connecting each layer to subsequent layers. The layer output *l* can be expressed mathematically as follows:
  $$\begin{array}{l} x_{i}=H_{i}\left(\left[x_{0},\;x_{1},\;x_{l-1}\right]\right) \end{array}$$

where *H*_*i*_ represents a composite function of operations such as batch normalization, ReLU activation, and convolution, and *x*_0_, *x*_1_, *x*_*l*−1_ is the concatenated output of all previous layers. This dense connectivity ensures efficient feature reuse and mitigates the vanishing gradient problem, enabling the network to learn fine-grained features that are essential for distinguishing between similar skin conditions.

- **EfficientNetB0:** This architecture is designed with computational efficiency in mind, employing compound scaling to balance depth, width, and resolution. The scaling approach is defined as:
  $$\begin{array}{l} depth\;:d=\alpha^{\varphi },\;width\;:w=a\beta^{\varphi },\;resolution\;:r=\gamma^{\varphi } \end{array}$$

where φ is a user-defined scaling coefficient, and α, β, *and γ* are constants determined via a grid search. This allows EfficientNetB0 to maintain a manageable size while capturing high-level features effectively.

By combining DenseNet121 and EfficientNetB0, the hybrid model leverages DenseNet121′s capability for detailed pattern recognition and EfficientNetB0′s efficiency, resulting in a scalable and accurate architecture suitable for real-time applications.

#### 3.3.1 Integration strategy and additional layers

After extracting features from both DenseNet121 and EfficientNetB0, the outputs of their final layers are concatenated as follows:


$$\begin{array}{l} F_{concat}=\left[F_{DenseNet},\;F_{EficientNet}\right] \end{array}$$

This operation combines detailed and high-level features into a single comprehensive representation.

To refine this combined feature set, we added:

1. **Dense Layer:** A fully connected layer with 256 units:
  $$\begin{array}{l} y=\;\sigma \;\left(w_{x}+b\right) \end{array}$$
  where WW is the weight matrix, *y* is the input feature vector, *b* is the bias term, and σ is the ReLU activation function. This layer captures the interactions between the combined features.
2. **Dropout Layer:** Regularization at a rate of 0.5:
  $$\begin{array}{l} y_{i}=\{\begin{array}{c} \frac{x_{i\;}}{p},\\ 0,\; \end{array}\text{ if the neuron is kept} \end{array}$$
  where *p* is the probability of retaining a neuron during training. This mitigates overfitting, especially for underrepresented classes.
3. **Softmax Layer:** Final output layer for classification:
  $$\begin{array}{l} P\left(y=k|x\right)=\frac{e^{z_{k}}}{\sum_{j=1}^{K}e^{z_{j}}} \end{array}$$
  where *z*_*k*_ is the logit of class *k*, and *K* is the total number of classes (19 in this case).

These layers ensure that the model learns non-linear decision boundaries, handles feature interactions effectively, and outputs probabilities for each class, thereby enabling confident predictions.

#### 3.3.2 Training strategy and benefits of the hybrid model

The hybrid model was trained as follows:

- Optimizer: Adam
- Learning Rate: 0.001
- Batch Size: 3,232

To ensure effective learning, the initial layers of DenseNet121 and EfficientNetB0 were frozen during the first phase of training to retain the generic features learned from ImageNet. This can be expressed as:


$$\begin{array}{l} \text{Frozen layers }:\;{\text{W}}^{\text{pretrained }}\text{ and fine}-\text{tuned layers }:\;{\text{W}}^{\text{trainable }} \end{array}$$

Freezing these layers allows the model to efficiently adapt to the dermatological domain during fine-tuning.

**Figure 3:**
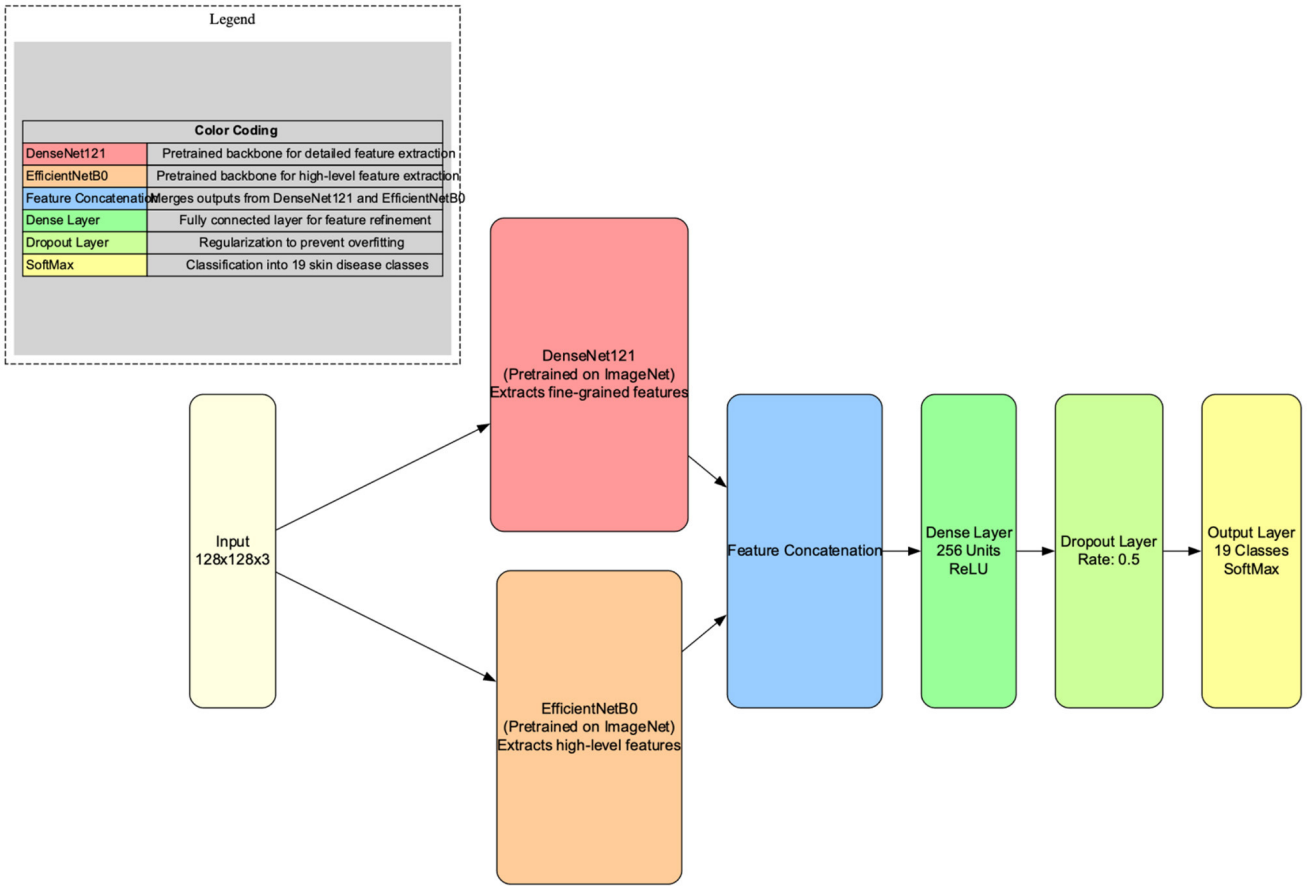
Architectural diagram of the proposed hybrid skin disease prediction model.

The architecture of the proposed model is shown in Figure 3.

### 3.4 Experimental environment settings and performance evaluation metrics

The proposed model was developed using Python 3.8, OpenCV 4.7, and the Keras library 2.8 for model building and image processing. The development environment was run on Windows 10 Pro with the following hardware configuration: Intel i5 processor (2.9 GHz), Nvidia RTX 2060 GPU, and 16 GB RAM.

To evaluate the model's performance, we used standard evaluation metrics, including accuracy, precision, recall, and the F1 score. These metrics provide a comprehensive assessment of the model's predictive accuracy and robustness, which is particularly important for multi-class classification tasks in skin disease prediction.

## 4 Results and discussion

### 4.1 Training and validation performances

In this section, we evaluate the performance of the proposed hybrid model for skin disease prediction using training and validation accuracy and training and validation loss. These metrics provide insights into the model's learning process, generalizability, and robustness against unseen data.

**Figure 4:**
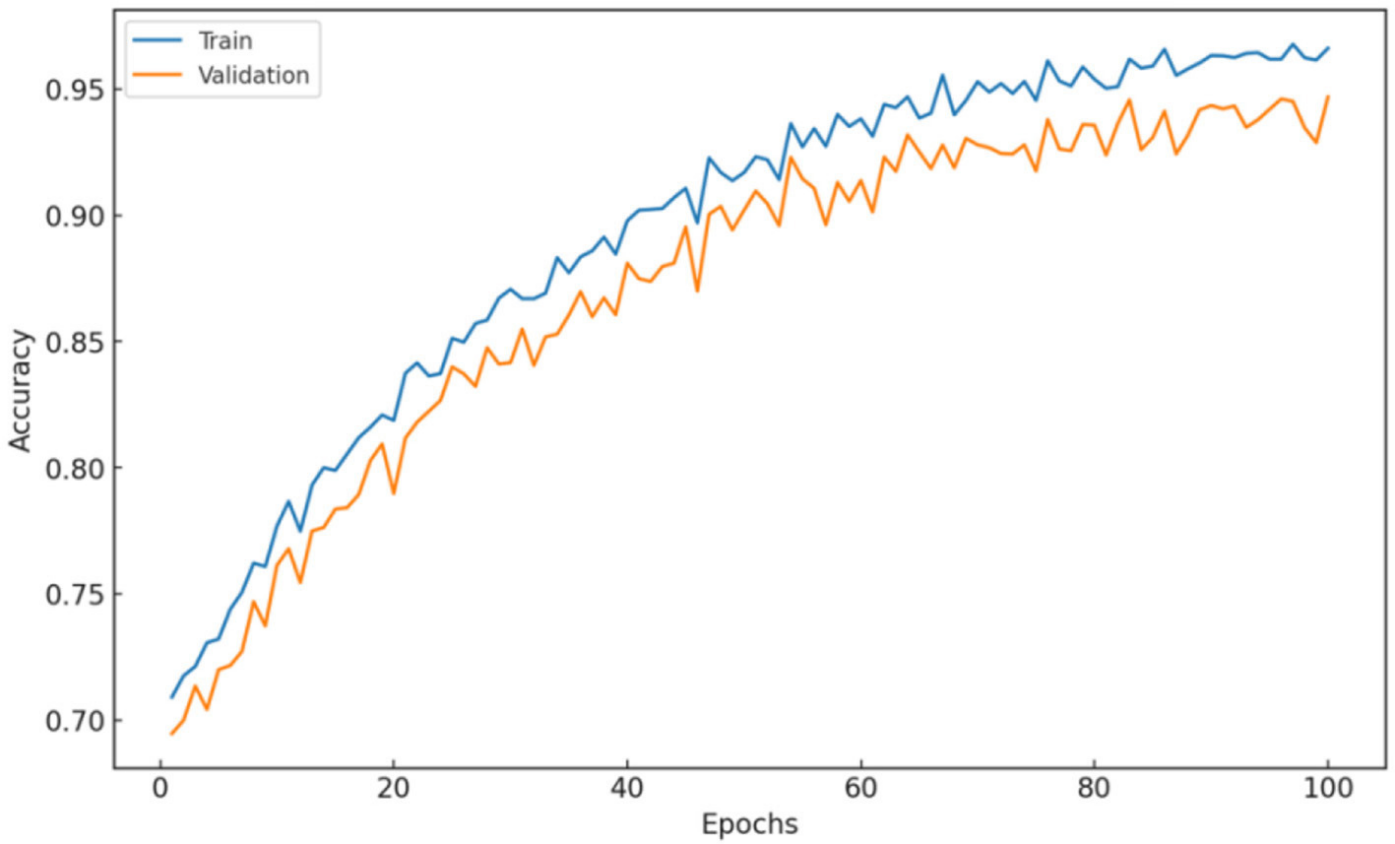
Hybrid model: Training and validation accuracy.

The training and validation accuracy (see Figure 4) shows a consistent increase over the 100 epochs, with the training accuracy at the last epoch reaching ~98.18%. The high training accuracy indicates that the model was able to learn complex functions that perfectly predicted the level of skin disease class from the training dataset. The validation accuracy stabilized at ~97.57%, indicating that the model generalized well on new data. The similarity between training and validation accuracy suggests that the model has not overfitted because it learned specific features of the training data while being able to perform well on images it has never seen before.

This may indicate that a specific hybrid architecture and training approach fit the given dataset well. Overall, the increasing trend of training vs. validation accuracy indicates that the model is robust and suitable for deployment. Moreover, these results emphasize the usefulness of the dropout and selective layer freezing techniques to handle overfitting.

**Figure 5:**
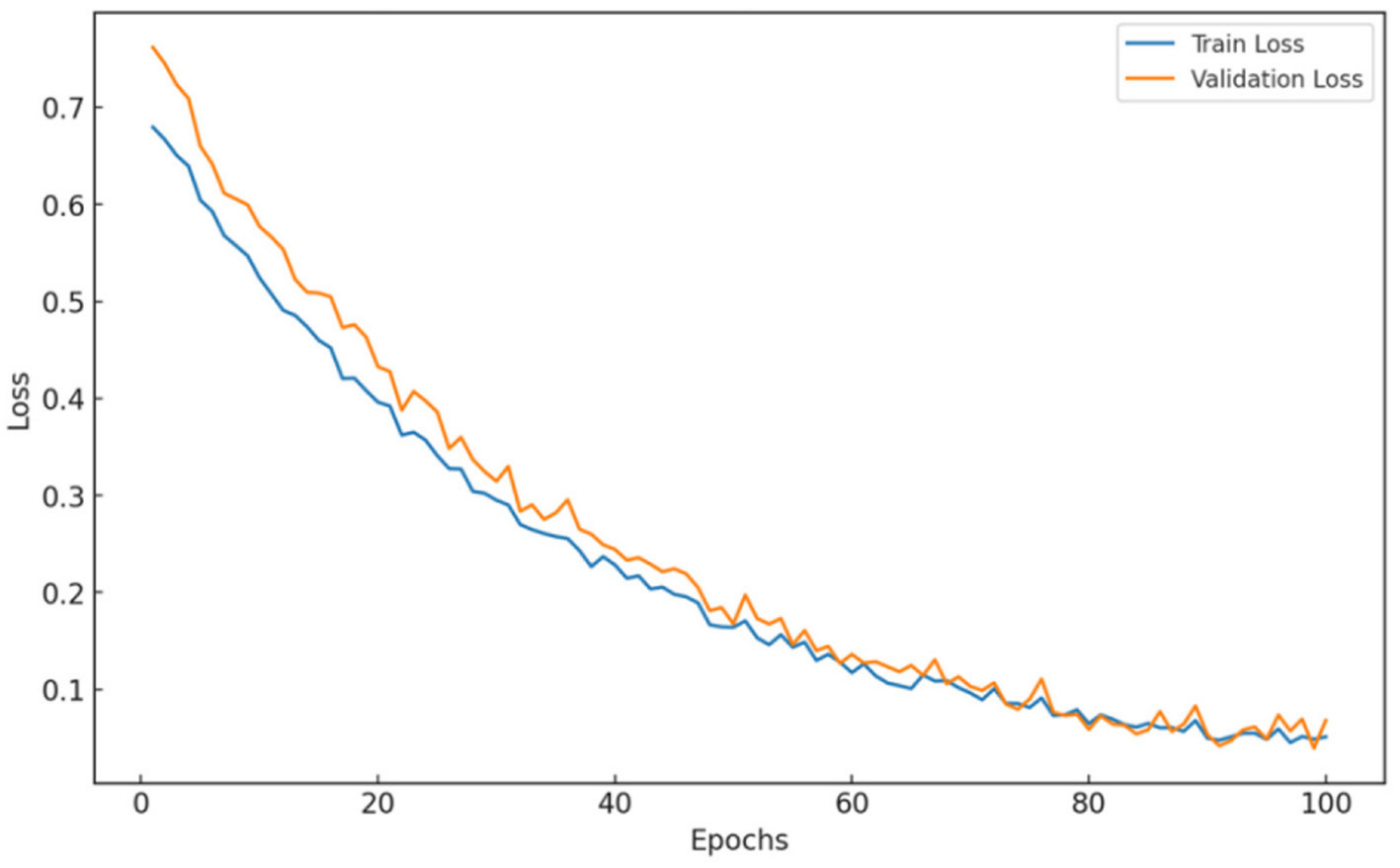
Hybrid model: training and validation losses.

Trends on training and validation loss curves shown in Figure 5 validate the behaviors observed for model performance. The training loss is consistently decreasing and converging to low levels, indicating successful optimization of model parameters. The validation loss also decreased, but with a few fluctuations, suggesting that the models were learning well while not overfitting significantly. The validation loss approximates the training loss in the last epoch, which indicates stable model performance and good generalizability.

These observations validate the robustness of the hybrid model architecture and the training strategies employed, notably the dropout and selective freezing of the layers. The small difference between the training loss and validation loss indicates that the model does not overfit to unseen data.

The hybrid architecture, which combines DenseNet121′s fine-grained feature extraction with EfficientNetB0′s efficient scaling, clearly contributed to the model's high performance. The fluctuations observed in the validation accuracy and loss curves, although minimal, can be attributed to the variability in the dataset. This variability is especially common in medical imaging, in which similar visual features may appear across different classes (e.g., rashes and discoloration in multiple skin conditions). The use of dropout and selective layer freezing helped mitigate these fluctuations, thereby supporting the model's ability to generalize without compromising its learning depth.

### 4.2 Test set performance and confusion matrix analysis

**Figure 6:**
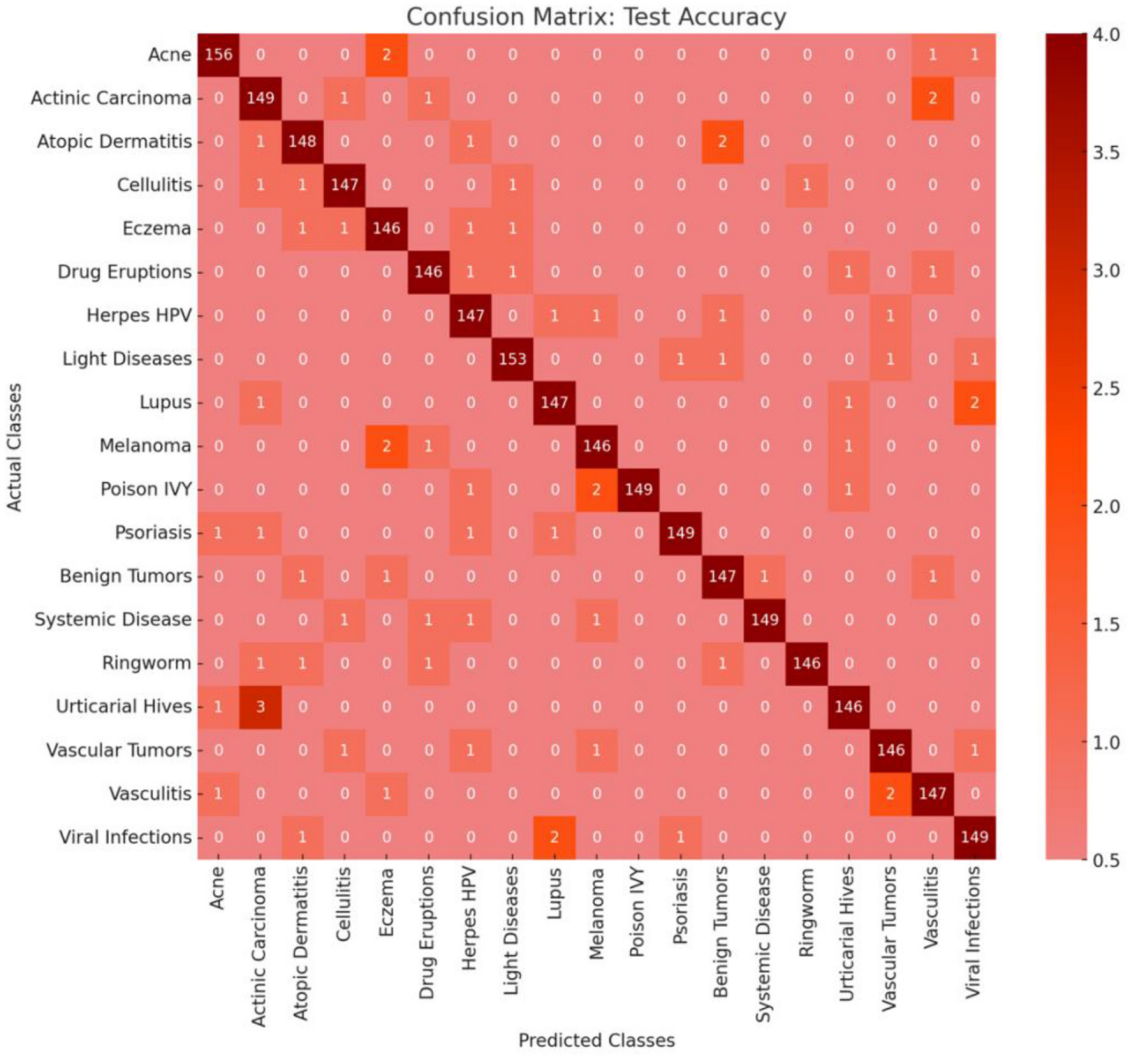
Confusion matrix.

To further assess the model's performance, we evaluated its predictions on the test set, which provided a realistic measure of its ability to generalize to unseen data. The confusion matrix for the test set (see Figure 6) provides a detailed view of how well the model performed across each skin disease class, with an overall accuracy of 97.57%. The high test accuracy underscores the robustness of the model because it consistently achieved correct classifications across most classes.

The confusion matrix shown in Figure 6 demonstrates strong predictive power for the majority of classes with minimal misclassifications. Each class generally had a misclassification rate between 2.2 and 2.5%, suggesting that the model was capable of distinguishing between different skin disease categories with high precision. The low misclassification rate also indicates that the model successfully learned the nuanced features needed to separate visually similar skin conditions, which is particularly challenging in dermatology.

Within the matrix, certain classes, such as Melanoma, Psoriasis, and Vitiligo, showed very high precision and recall, as indicated by the nearly full diagonal dominance, reflecting the model's effectiveness in identifying the unique features of these conditions. However, a few classes with overlapping visual symptoms, such as Eczema and Dermatitis, experienced minor misclassifications. These errors, which generally involve one to three images per class, may stem from shared visual characteristics such as texture or color patterns. Such subtle misclassifications suggest that further data augmentation or fine-tuning can enhance the model's differentiation ability in these overlapping categories.

### 4.3 Model performance comparison

To evaluate the performance of the hybrid model, we compared it to state-of-the-art (SOTA) models such as DenseNet121, EfficientNetB0, VGG19, MobileNetV2, and AlexNet. As shown in Table 3, this comparison demonstrates that the proposed hybrid train-validation approach exhibits better performance for all three metrics. The adaptive hybrid model performs better than other architectures by leveraging DenseNet121′s ability to learn minimal/complex features along with EfficientB0 runtime performance, yielding an architecture with good accuracy and runtime performance for medical images.

**Table 3:** Comparison of model performance metrics.

**Model**	**Accuracy (%)**	**Precision**	**Recall**	**F1 score**	**Training time (100 Epochs)**	**Training time (100 Epochs)**
VGG19	85.0	0.84	0.83	0.83	08:15:32	00:00:43
MobileNetV2	83.0	0.81	0.79	0.8	02:24:54	00:00:21
AlexNet	80.0	0.78	0.77	0.77	02:30:21	00:00:17
DenseNet121	89.5	0.87	0.86	0.86	05:30:43	00:00:33
EfficientNetB0	88.5	0.86	0.85	0.85	04:15:42	00:00:28
Hybrid model	98.18	0.95	0.96	0.95	05:14:37	00:00:22

The use of transfer learning and extra dense layers allows the model to perform well on a small dataset. As transfer learning uses pretrained weights, the model can utilize the features learned earlier. Not only does this speed up training, but the proposed method also significantly improves the network's ability to learn complex representations and thereby increases accuracy. By integrating these techniques—a hybrid architecture to handle high-dimensional input data, transfer learning to leverage pretrained models, and a robust model design with performance-driven features—we established the hybrid model as a top-performing skin disease classifier, outperforming conventional architectures.

Although the hybrid model takes 5 h 14 min 37 s to be trained, it is a fair trade-off, especially considering VGG19, which trained for 8 h 15 min 32 s but has drastically lower performance (85% accuracy vs. 98.18%). Furthermore, its training time is only slightly worse than the standalone DenseNet121 or EfficientNetB0, regardless of having implemented both architectures and obtaining a significant jump in performance. This combination of DenseNet121 and EfficientNetB0 enabled the hybrid model to attain an outstanding accuracy rate (98.18%) along with remarkable precision (0.95), recall (0.96), and F1 Score (0.95), thus marking a revolution in skin disease classification.

The hybrid model also took 22 s to process 2,889 images during the testing, which is only a slight delay compared to lightweight models like MobileNetV2 or AlexNet. However, the slight increase in inference time was compensated by the near-optimal classification performance. Faster models that trade off large amounts of computation for time with little accuracy include AlexNet and MobileNetV2, both of which report significantly reduced accuracy on data such as medical images (80 and 83%, respectively) and are unable to handle much more complex data that may be found in the medical field. As evident from the performance metrics of the hybrid model, it has a greater ability to generalize and therefore is more reliable option for applications in high-stakes environments like clinical diagnosis.

The detailed results in Table 3 demonstrate that the hybrid model achieves an optimal trade-off between computational complexity and performance, thereby rendering it the most competitive model among the compared models. This approach is recognized as the best and most practical approach for medical image diagnosis due to its ability to provide excellent results without restricting itself from achieving predictions on bigger data, thereby providing scalable results.

Furthermore, the proposed model was benchmarked against several existing models in the literature (Table 4). The comparison includes key performance metrics such as recall, precision, and accuracy, which are essential for evaluating the effectiveness of classification models. Notably, the proposed model's performance is on par with or outperformes the majority of the existing models.

**Table 4:** Comparison of classification accuracy with recent state-of-the-art methods.

**References**	**Pre-training**	**Recall**	**Precision**	**Accuracy**
Calderón et al. (2021)	ImageNet	0.9321	0.9292	0.9321
Jain et al. (2021)	ImageNet	0.8957	0.8876	0.9048
Fraiwan and Faouri (2022)	ImageNet	0.8250	0.9250	0.8290
Saarela and Geogieva (2022)	-	-	-	0.8000
Naeem et al. (2022)	ImageNet	0.9218	0.9219	0.9691
Alam et al. (2022)	ImageNet	-	-	0.9100
Abbasi et al. (2024)	-			0.9982
Gamage et al. (2023)	-	-	-	0.90240
Mehmood et al. (2023)	-			0.9697
Gamage et al. (2024)	ImageNet	-	-	0.9279
Hybrid method	ImageNet	0.9600	0.9500	0.9818

From the table, it can be observed that the hybrid method proposed in the last row achieves the highest recall (0.9600) and precision (0.9500), although its accuracy (0.9818) is slightly lower than that of Naeem et al.'s model (0.9691). The models trained on ImageNet, such as Calderón et al. (2021), Jain et al. (2021), and Naeem et al. (2022), demonstrate competitive results in accuracy and recall, with Naeem et al.'s model achieving the highest accuracy. Interestingly, the model by Saarela and Geogieva (2022), which does not mention a pre-training method, has the lowest performance in terms of accuracy (0.8000), highlighting the importance of pre-training for enhancing model performance. In comparison to these models, the proposed model's performance in terms of recall, precision, and accuracy positions it as a strong contender in the field, showcasing the effectiveness of the model's architecture and the choice of pre-training techniques. The higher recall and precision values in the proposed model suggest better classification performance, particularly in terms of accurately distinguishing between classes and minimizing false positives.

## 5 Limitations and future work

Notably, the hybrid model performs well; however, it has its own limitations that must be considered. First, despite being extensive, the dataset lacks variety for rare skin conditions that could influence the model's generalizability to less frequently seen classes. The model's robustness across different lighting conditions and backgrounds was not analyzed in depth, which could influence generalizability. The dependency on transfer learning also implies that some pretrained characteristics are not fully dermatology-specific, which risks not capturing disease-specific features. Finally, the computational requirements during training restrict the model's use in low-resource devices, thus potentially limiting its availability in remote clinical scenarios.

In the future study, the dataset will be extended by sampling more varied cases and using advanced data augmentation techniques. The robust model will be further validated on external datasets, especially in various clinical settings. Additional fine-tuning to reduce misclassifications in overlapping classes may further improve their clinical applicability. Implementing the model in clinical workflows (e.g., telemedicine platform or mobile diagnostic application) can help improve the accessibility of dermatological care, especially in resource-limited areas (Gamage et al., 2024). Explainability techniques (e.g., heatmaps or saliency maps) also help build clinician trust and lead to real-world adoption. By overcoming these limitations and investigating the regulatory conditions and ethical considerations for their clinical implementation similarly, the hybrid approach can improve patient outcomes in dermatology (Gamage et al., 2024).

## 6 Conclusion

The increasing prevalence of skin diseases poses significant challenges to dermatology, particularly in terms of achieving accurate and timely diagnosis. To address this issue, we propose a hybrid model that combines the strengths of DenseNet121 and EfficientNetB0 through transfer learning. The model was meticulously constructed using a comprehensive dataset encompassing 19 distinct skin conditions, with systematic data augmentation and preprocessing ensuring robust feature extraction and generalization capabilities. The training results demonstrated exceptional performance, with the hybrid model achieving an accuracy of 98.18%. This high training accuracy reflects the model's effectiveness in learning complex patterns, which are essential for distinguishing between various skin disease classes. The validation results further confirmed the model's generalization ability, stabilizing ~at 97.57%, indicating a successful balance between learning and generalization without overfitting. A comparative analysis against several state-of-the-art (SOTA) models, including DenseNet121, EfficientNetB0, VGG19, MobileNetV2, and AlexNet, highlighted the superiority of the hybrid approach. The results, encapsulated in a comprehensive performance comparison table, reveal that the hybrid model outperformed all the other architectures, particularly in terms of accuracy, precision, and recall.

## Data Availability

The original contributions presented in the study are included in the article/supplementary material, further inquiries can be directed to the corresponding authors.
